# Health service delay among pulmonary tuberculosis patients presenting to a National Referral Hospital, Kampala, Uganda: a cross sectional study

**DOI:** 10.11604/pamj.2013.15.84.2692

**Published:** 2013-07-02

**Authors:** Catherine Kansiime, Stephen M Kiwuwa, Mugenyi Levi, Benon B Asiimwe, Achilles Katamba

**Affiliations:** 1 Department of Health Policy Planning and Management, School of Public Health, College of Health Sciences, Makerere University, P. O Box 7072, Kampala; 2 Clinical Epidemiology Unit, School of Medicine, College of Health Sciences, Makerere University, P. O Box 7072, Kampala; 3 Infectious Disease Research Collaboration, Mulago Hospital Complex, P.O Box 7475, Kampala; 4 Department of Medical Microbiology, College of Health Sciences, Makerere University, P. O Box 7072, Kampala

**Keywords:** Tuberculosis, health service delay, treatment delay, diagnostic delay, pulmonary tuberculosis

## Abstract

**Introduction:**

Delay in the diagnosis of pulmonary tuberculosis (PTB) is common in many countries in Sub-Saharan Africa. Timely diagnosis of active tuberculosis is crucial in minimizing morbidity and mortality in the community as well as nosocomial transmission in health care facilities. This study aimed at determining factors associated with health service delay in the diagnosis and initiation of treatment among new PTB patients presenting to the National Referral Hospital-Mulago.

**Methods:**

This was a cross-sectional study among eligible new PTB patients presenting at the National referral TB treatment center Mulago hospital, between March to May 2009. The patients were consecutively recruited and interviewed using a semi-structured questionnaire to assess socio- demographic and health service factors. Multivariate logistic regression using odds ratios and 95% confidence intervals was done.

**Results:**

Two hundred and sixty six newly diagnosed PTB patients were enrolled, of which 65.4% experienced health systems delay. The median health service delay was 9days (IQR=8-19). Factors associated with health service delay were: 1n-patient (OR= 4.68, 95% CI: 1.91-11.45), secondary as highest level of education attained (OR= 3.56, 95% CI: 1.18-10.74), primary as highest level of education attained (OR= 6.70, 95% CI: 2.13-21.02), presence of fever (OR= 3.28, 95% CI: 1.05-10.79), and patient delay at health facility (OR= 5.01, 95% CI: 1.33-18.9).

**Conclusion:**

The study found a significant proportion of Health service delay among pulmonary tuberculosis patients presenting at the referral hospital. Being an in-patient and having fever as a symptom of tuberculosis needs further attention in order to have timely diagnosis. There is need for awareness on TB especially that most of the TB symptoms present like other febrile illnesses such as malaria and needs consideration when patients present to a health facility.

## Introduction

World Health Organization (WHO) estimates that 9.2 million people develop TB annually, and developing countries account for 95% of TB infected persons worldwide [1]. Globally, the burden of TB is escalating, but a significant problem lies with the fact that many cases remain undiagnosed. This could be due to a number of factors, principally found within the categories: patients delay and health service delay. The Lake Victoria basin in Sub-Saharan Africa is one of the regions with the highest burden of TB [2].

Early diagnosis of tuberculosis and prompt initiation of treatment is essential for an effective control programme. It is estimated that the average incidence of tuberculosis in sub-Saharan African countries more than doubled between 1990 and 2005, from 149 to 343 per 100,000 population, whereas other regions saw stable or declining rates [3]. Delay in the diagnosis may worsen the disease in terms of morbidity and mortality in the communities as well as the health care facilities, enhance tuberculosis transmission in the community and therefore, patients′ awareness to tuberculosis symptoms together with health workers′ ability to diagnose the disease are important factors to control the spread of the infection in a community [4].

Previous studies [5, 6] focused on patient and community factors (health seeking behavior) that influence delay in diagnosis and initiation of treatment with little emphasis on the delay that occurs once a symptomatic patient makes contact with the health care system. Health system delay was defined as the interval from the first medical visit with symptoms suggestive of PTB to the date tuberculosis is diagnosed and treatment initiated [7]. This study aimed at assessing the factors associated with health service delay in the diagnosis and initiation of treatment at a TB referral centre in a high disease burden setting. This knowledge may inform the implementation of measures for timely diagnosis and treatment so as to reduce transmission of TB in the health facilities and the communities.

## Methods

### Study design and setting

Between March and May 2009, a cross-sectional study to assess factors associated with health service delay in the diagnosis and initiation of TB treatment was done among patients with PTB attending the National Tuberculosis Referral Center (NTRC), Kampala city, Uganda. The NTRC offers free TB treatment. Mulago referral hospital is the principal facility providing in-patient TB care in the country. Diagnosis and management of Tuberculosis is based on the WHO recommended DOTS strategy with case detection through predominantly passive case detection and directly observed, standardized, free-of-charge short-chemotherapy regimen with recommended regimens. It has an in-patient capacity of 100 beds and manages in-patients and outpatient TB patients referred from in-patient wards in Mulago hospital, regional referral hospitals and out-patient services from both government and non-government health facilities in and outside Kampala Capital city. The TB clinic operates three days a week (Monday, Wednesday, and Thursday) and attends to approximately 490 TB patients per month as depicted in the TB register of 2008. Of the 490 TB patients, about 100 are new PTB suspects with both smear positive and negative sputum samples.

### Participant selection

All PTB patients 18 years and above presenting at the NTRC within two weeks of diagnosis or initiation of TB treatment were eligible for enrollment. Consenting eligible patients were consecutively enrolled on clinic days. In order to avoid recall bias, relapses, retreatment cases as well as patients who were very sick were excluded. Both in-patient and out patients were studied.

### Data Collection

Using interviewer administered questionnaires, data were collected on socio-demographic variables of the patients (age, sex, occupation, marital status, and level of education of the respondents), clinical data (sputum smear status, HIV status, TB symptoms), and health service factors such as health facility first visited by the patients, hospitalization status, tests done such as sputum smears and chest x-ray, availability of TB drugs as well use of non anti-tuberculosis drugs. The questionnaire was pre-tested among 30 TB patients prior to data collection. The Principal Investigator trained two research assistants (one nurse and one laboratory technician) on how to collect data and briefed them on the objective of the study. Interviews were administered in the main local language spoken in the study setting which is Luganda. Other data sources such as patient treatment cards were used to capture information on sputum smear status, date when diagnosis was made and date of initiation of TB treatment.

Health service delay was defined as the interval from the first medical visit to the date TB was diagnosed and treatment initiated [7]. In this study, health service delay was defined as an interval of more than five and 10 days for smear positive and smears negative patients respectively from the day a TB suspect presented to the health facility with symptoms suggestive of TB to initiating TB treatment (guidelines from the National Tuberculosis and Leprosy Control Programme). Smear-positive tuberculosis is considered if any of the two sputum smear examinations is positive whereas sputum smear negative is considered when: At least 2 sputum specimens negative for AFB and Radiological abnormalities consistent with active TB disease and Non-response to a course of broad spectrum antibiotics (excluding fluoroquinolones) for seven days and decision by a clinician to treat as pulmonary TB with a full course of anti-TB drugs. Health service delay was analyzed as a combination of delay in diagnosis and initiation of treatment; therefore, for one to have had health service delay, he or she would have delayed at both diagnosis and initiation of treatment or at any of the two points. The two were combined basing on the above definition of health service delay because the factors that lead to delay in treatment may be influenced by delay in diagnosis or the other way round.

### Data management and analysis

On a daily basis, the principal investigator collected and checked the completed forms and corrected errors that arose during data collection. To ensure quality, collected data were double entered into Epi Data Entry version 3.1 and validated. Records that were discordant during the validation process were resolved by consulting the questionnaire. Data were exported to STATA version 10 for analysis. Health service delay was dichotomized into no health service delay and health service delay using the cut offs of more than 5 and 10 days for smear positives and smear negatives respectively.

Categorical variables were summarized into frequencies and percentages, the Chi square were used for analyzing categorical data. Continuous variables were summarized into ranges, means and medians. Variables with P-value≤0.2 at bivariate level were considered for the multivariable analysis for both smear negative and smear positive PTB patients. Multivariable logistic regression was used to assess the factors independently associated with health service delay in diagnosis and initiation of treatment using odds ratios (ORs) and 95% confidence intervals (CIs). A variable was considered a confounder if the crude and adjusted ORs differed by more than 10%. A p value ≥ 0.05 was considered statistically significant.

### Ethical considerations

Study approval was sought from Makerere University Clinical Epidemiology Unit and the National Tuberculosis Referral Center, while ethical approval was sought from the Makerere School of Medicine Research and Ethics Committee. Written informed consent was obtained from the patients and confidentiality was kept by using patient identification numbers rather than names. The data collected was kept under lock and key before and after data entry.

## Results

Two hundred and sixty six (266) eligible adults newly diagnosed with smear positive (207, 78%) and smear negative (59, 22%) PTB were recruited in the study. The median age was 34 years with inter-quartile range of 26-42. There were equal numbers of male and female respondents, a majority of whom (140, 52.6%) had attained primary education. Primary education takes seven years, secondary six years and tertiary a minimum of four years, hence total education is seventeen years. Details of other socio-demographic characteristics of the participants are shown in Table 1.

**Table 1:** Socio-demographic characteristics of 266 PTB respondents presenting to Mulago hospital, 2009

Variables	Frequency N(266)+	Percent (%)
**Age**		
≤33	127	47.8
≥34	139	52.2
**Sex**		
Male	133	50.0
Female	133	50.0
**Education**		
Tertiary	38	14.3
Secondary	88	33.1
Primary	140	52.6
**Occupation**		
Civil servant	41	15.4
Businessman/woman	115	43.2
Farming/domestic	24	9.0
Others*	86	32.3
**Marital status**		
Married	175	65.8
Unmarried	91	34.2
**Religion**		
Christian	146	54.9
Muslim	120	45.1

* Others included students, builders, mechanics, driver and carpenter.

+ The total number of 266 includes; both smear negative and positive participants.

### Symptoms suggestive of TB at the time of presentation to the health facility

Cough was the main symptom, reported by (254, 95.5%) patients, fever by (229, 86.1%), night sweats by (220, 82.7%), chest pain (206, 77%), appetite loss (203, 76.3%). Only (52,19.6%) of the participants had blood in sputum, which is usually seen in cases of advanced TB, while a few respondents mentioned diarrhoea (94, 35.3%), neck swellings (82, 30.8%) and weight loss (93, 34.9%).

### Distribution of health service factors among respondents

Respondents were asked questions related to health service factors that could have led to a delay in the diagnosis and initiation of anti-TB treatment. Factors such as hospital admission, sputum smear status and first point of contact were assessed. Furthermore, first experiences at the hospital such as prescription of non anti-TB medication, performance of sputum smear test, chest x-ray, ward of admission, and first examiner at health facility were assessed. Details are shown in Table 2.

**Table 2:** Distribution of health service factors of 266 PTB respondents presenting to Mulago hospital, 2009

Health Service Factor	Frequency N(266)+	Percent (%)
**Hospital admission status**		
Outpatient	127	47.7
Inpatient	139	52.3
**Sputum smear status**		
Positive	207	77.8
Negative	59	22.2
**1** ^**st**^ **point of contact**		
Hospital	210	79.0
Private clinic/dispensary/pharmacy	46	17.3
Traditional healer	10	3.7
**First experience at the hospital**		
Referral		
No	223	84.1
Yes	42	15.9
**Given non-TB medication**		
No	142	53.6
Yes	123	46.4
**Sputum smear test**		
No	82	30.9
Yes	183	69.1
**Chest x-ray scan**		
No	105	39.6
Yes	160	60.4
**Admitted**		
Yes	145	54.5
No	121	45.5
**Ward admitted**		
Surgical	31	21.8
Medical	110	75.9
TB ward	4	2.8
**First examiner at the hospital**		
Doctor	228	85.7
Nurse/clinical officer	38	14.3

* others included; traditional healers and herbal treatment clinics.

+ The total number of 266 includes; both smear negative and positive participants.

### Distribution of delays among the respondents

The median health service delay was nine days with a inter-quartile range (IQR=8-19). Median diagnostic delay was 6days (IQR=8-12) and treatment delay was 3days (IQR=5-7). Of the 266 respondents (174, 65%) experienced health system delay, of these (127, 73%) were smear positive and (47, 27%) were smear negative, whereas, (92, 35%) did not experience health system delay and of these (80, 87%) were smear positive and (2, 13%) were smear negative.

### Factors associated with health service delay at bivariate analysis

Factors significantly associated with health service delay at bivariate analysis were primary education (OR=2.42, 95% CI: 1.17-5.03), in-patients (OR=1.72, 95% CI: 0.35-0.97), negative sputum smear (OR=2.47, 95% CI: 1.23-4.90), prescription of non-anti-tuberculosis treatment (OR=1.69, 95% CI: 1.01-2.82) was done at presentation to the health facility before diagnosis of PTB, and patient delay as a reason for health service delay (OR=3.05, 95% CI: 0.98-9.48). Variables that were ≤5.02 at bivariate were considered for multivariable analysis as depicted in Table 3.

**Table 3:** Association between socio-demographic characteristics, symptoms, health service factors and health service delay at bivariate analysis among 266 PTB respondents.

Variable	No delay n=92 (%)	Delay n=174 (%)	Unadjusted OR	95%CI	*P* –value
**Sex**					
Male	52 (39.1)	81 (60.9)	1.00		
Female	40 (30.1)	93 (69.9)	1.49	0.90 – 2.48	0.12*
**Education**					
Tertiary	20 (52.6)	18 (47.4)	1.00		
Secondary	28 (31.8)	60 (68.2)	2.38	1.09 - 5.19	0.03*
Primary	44(31.4)	96 (68.6)	2.42	1.17 – 5.03	0.02*
**Fever**					
No	8(21.6)	29(78.4)	1.00		
Yes	84(36.7)	145(63.3)	2.10	0.92 – 4.80	0.08
**HIV status**					
Positive	49(37.7)	81(62.3)	1.00		
Negative	36(29.3)	87(70.7)	1.46	0.86 – 2.47	0.16
Do not know	7(53.9)	6(46.1)	0.52	0.16 – 1.163	0.26
**Admission status**					
Outpatient	52(40.9)	75(59.1)	1.00		
In-patient	40(28.8)	99(71.2)	1.72	1.03-2.86	0.04*
**Smear Status**					
Positive	56(27.1)	151(72.9)	1.00		
Negative	36(61.0)	23(39)	2.47	1.23 – 4.90	0.01*
**Non-TB medication**					
No	57(40.1)	85(59.9)	1.00		
Yes	35(28.5)	88(71.5)	1.69	1.01 – 2.82	0.05*
**Reasons for delay**					
Not clinic days	13 (48.1)	14(51.9)	1.00		
Irregular TB drugs	25(42.4)	34(57.6)	1.26	0.51 – 3.15	0.62
Laboratory delays	18(31.0)	40(68.9)	2.06	0.81 – 5.27	0.13
Patient delays	7(23.3)	23 (76.7)	3.05	0.98 – 9.48	0.05*

† Others included, students, builders, mechanic, driver and a carpenter; Those who did not delay and those who delayed include both smear positive and smear negative participants.

* Significant P-value ≤0.05;. OR = Odds Ratio; CI = Confidence Interval and variables with P-value ≤0.2 that were taken for multivariate analysis.

### Factors associated with health service delay at multivariable analysis

Respondents who presented with fever were 3.28 times more likely to have delayed than those who did not have fever; Participants who were in-patient were 4.68 times more likely to have delayed at the health system than out-patients. Respondents who mentioned patient delay as a reason for health service delay were 5.01 times more likely to delay than those who mentioned other health service related reasons. Respondents who attained secondary and primary were 3.56 times and 2.13 times more likely to have delayed than those who attained tertiary education. The majority of the respondents who had attained secondary education had completed senior two (education of nine years). These were the only factors found to be independently associated with health service delay after controlling for the extraneous factors (Table 4). There was no interaction and no confounding between the predictor variables and health service delay.

**Table 4:** Multivariate analysis of factors associated of health service delay among 266 PTB respondents

Variable	Unadjusted OR	95%CI	Adjusted OR	95%CI	P-value
**Admission status**					
Out -patient	1.00		1.00		
In-patient	1.72	1.03-2.86	4.68	1.91-11.45	0.001*
**Education**					
Tertiary	1.00		1.00		
Secondary	2.38	1.09 - 5.19	3.56	1.18 – 10.74	0.02*
Primary	2.42	1.17 – 5.03	6.70	2.13 – 21.02	0.01*
**Fever**					
No	1.00		1.00		
Yes	2.10	0.92-4.80	3.28	1.05 – 10.79	0.05*
**Reasons for health service delay**					
Public holidays	1.00		1.00		
No TB drugs	1.26	0.51 – 3.15	0.92	0.34 – 2.51	0.87
Lab delays	2.06	0.81 – 5.27	1.63	0.60 – 4.47	0.34
Patient delays	3.05	0.98 – 9.48	5.01	1.33 – 18.9	0.02*

* Significant P-value ≤0.05. OR = Odds Ratio; CI = Confidence Interval

## Discussion

### Health service delay in diagnosis and treatment of TB

In this study, we found high health service delay where, 174 (65.4%) of the study participants were found to have experienced delay by the health care system. An earlier study conducted in Mulago hospital [5], showed that 74% of delay in receiving TB services was due to health service delay. However, health service delay is still high indicating limited impact on interventions put in place to improve timely health care delivery since 2005. In this study, the median health service delay was 9 days, which is slightly higher than a study done in Ethiopia which found a median health service delay of 6 days [8]. However, this was much lower than median health service delays of 19 days in Estonia [9]. The low median delay may be due to the cut off set for this study that was interested in delay at the health system.

In this study, we found high delay (75.2%) in initiation of TB treatment more than delay in diagnosis. This could be due to insufficient and irregular supply of TB drugs in Uganda during the course of this study. However, this difference was not statistically significant during multivariable analysis. In contrast, a study done in Rwanda [10] reported 90% of the patients were started on treatment within 24 hours of diagnosis with a median treatment delay of one day. There is need for consistence in the supplyvailability of anti-tuberculosis drugs in order to reduce transmission in health facilities and communities as well as reduce cases of multi-drug resistence TB.

### Socio-demographic and clinical factors associated with health service delay

Participants who had attained primary as the highest level of education were 6.70 times more likely to delay than those who attained tertiary education; participants who attained secondary education (senior two which is 9 years) were 3.56 times more likely to delay than those who attained tertiary as the highest level of education. A study done in Zambia found that lower education of less than 9 years was a risk factor of health service delay [11]. Although education level was the only socio-demographic factor that was found statistically significant, other variables were not. However, a study in Pakistan [12] found delays in females but no statistical significance by age, education, occupation and smoking.

In this study, participants who had fever were 3.28 times more likely to have delayed than those who did not have fever. This could be because some of the symptoms of tuberculosis of which fever is included is similar to other febrile illnesses such as malaria, typhoid and brucellosis hence resulting in misdirected treatments and underreporting [13, 14]. Whereas, in France [15] initial fever was associated with reduced patient delay. At bivariable analysis patients who were started on non-tuberculosis treatment were associated with health service delay but this association was not significant at multivariable analysis.

### Health service factors

Factors significantly associated with health service delay in this study were admission status where in-patient were 4.68 times more likely to delay than those who were out-patients. In-patients were those respondents who had been admitted in other wards such as Medical before being referred to the TB wards 5 and 6. In-patients on TB wards 5 and 6 were not included in the study because they were retreatment patients and or those resistant to TB treatment. This may explain the fact that first admission to other wards could have influenced health service delay. However, a study in Zambia also found that outpatient diagnosis of tuberculosis was associated with health service delay [11] which they attributed to patients? of seeking care from numerous health care providers before visiting a hospital.

Participants who mentioned patient delay as a reason for health service delay were 5.01 times more likely to have delayed than those who mentioned other factors for health service delay. Reasons for patient delay included; transport costs and distance to health facility. However, no association was found between the point of first contact (health facility) with symptoms suggestive of TB and health service delay. This could be because the majority (79%) of the patients in this study reported first to a hospital. This suggests that most of the respondents were informed and knowledgeable about the importance of visiting a hospital for medical care. Similarly, a study done in South Africa [6] found that most patients (75%) used the public health system as the first point of contact. There was also no association between the specialty of first health care provider (examiner) contacted by the patient at first presentation at a health facility. A similar finding was recorded in Estonia in which there was no association between speciality of first health care provider contacted by the patient [9].

### Strengths and weaknesses of the study

Unlike a previous study [5] in Uganda that focused mainly on the patient factors that determine delay in diagnosis and initiation to treatment, the current study focused on the factors that determine health service delay in the diagnosis and initiation of treatment. All interviews were performed within less than a month of diagnosis and treatment of PTB, ensuring that the patients remembered the course and duration of diagnosis, hence minimizing errors of recall bias. The study was not population based, but facility-based covering one referral hospital that receives TB patients from all over the country. The study design focused only on the quantitative method, we recommend a study in future to conduct a detailed qualitative study through key informant interviews with the health providers In order to capture in depth information on health systems delay.

## Conclusion

The study found a significant proportion of Health service delay among pulmonary tuberculosis patients presenting at the referral hospital. Being an in-patient and having fever as a symptom of tuberculosis needs further attention in order to have timely diagnosis. There is need for awareness on TB and emphasis that most of TB symptoms present like other febrile illnesses such as malaria and this needs to be considered when patients present to a health facility. On the other hand, health service delay was not associated with first health point of contact and health care provider who examined the patient on first presentation to the health facility.

### Recommendation

In future, s study using qualitative methods focusing on the health system should be conducted in order to obtain detailed information on the health service factors that influence health system delay in the diagnosis and treatment of tuberculosis for better control of TB in the health system.
